# Experiences and factors associated with transphobic hate crimes among transgender women in the San Francisco Bay Area: comparisons across race

**DOI:** 10.1186/s12889-021-11107-x

**Published:** 2021-06-02

**Authors:** Akua O. Gyamerah, Glenda Baguso, Edda Santiago-Rodriguez, Aria Sa’id, Sean Arayasirikul, Jess Lin, Caitlin M. Turner, Kelly D. Taylor, Willi McFarland, Erin C. Wilson, Paul Wesson

**Affiliations:** 1 Center for AIDS Prevention Studies, Department of Medicine, University of California, San Francisco, California USA; 2 Department of Community Health Systems, School of Nursing, University of California, 2 Koret Way, San Francisco, CA 94143 USA; 3 Transgender District, San Francisco, USA; 4 San Francisco Department of Public Health, Trans Research Unit for Equity, San Francisco, USA; 5 Department of Public Health, Center for Public Health Research, San Francisco, USA; 6 Institute for Global Health Sciences, University of California, San Francisco, USA; 7 Department of Epidemiology & Biostatistics, University of California, San Francisco, USA

**Keywords:** Transgender women, Hate crimes, Violence, Transphobia, Gender-based violence

## Abstract

**Background:**

Trans women experience high rates of gender-based violence (GBV)—a risk factor for adverse health outcomes. Transphobic hate crimes are one such form of GBV that affect trans women. However, little is understood about factors that shape transphobic hate crimes and racial/ethnic variation in these experiences. To contextualize GBV risk and police reporting, we examined self-reported types and correlates of transphobic hate crimes by racial/ethnic group of trans women in the San Francisco Bay Area.

**Methods:**

From 2016 to 2018, trans women participated in a longitudinal cohort study of HIV. Secondary data analyses (*N* = 629) examined self-reported experiences of transphobic hate crimes (i.e., robbery, physical assault, sexual assault, and battery with weapon) by race/ethnicity, and whether hate crimes were reported to the police. Chi-square tests and simple logistic regression examined demographic, sociocultural, and gender identity factors associated with transphobic violence experiences and police reporting.

**Results:**

About half (45.8%) of participants reported ever experiencing a transphobic hate crime; only 51.1% of these were reported to the police. Among those who reported a hate crime experience, Black (47.9%) and Latina (49.0%) trans women reported a higher prevalence of battery with a weapon; White (26.7%) and trans women of “other” race/ethnicities (25.0%) reported a higher prevalence of sexual assault (*p* = 0.001). Having one’s gender questioned, history of sex work, homelessness as a child and adult, and a history incarceration were associated with higher odds of experiencing a transphobic hate crime. Trans women who felt their gender identity questioned had lower odds of reporting a hate crime to the police compared to those did not feel questioned.

**Conclusions:**

A high proportion of trans women experienced a transphobic hate crime, with significant socio-structural risk factors and racial differences by crime type. However, crimes were underreported to the police. Interventions that address structural factors, especially among trans women of color, can yield violence prevention benefits.

## Background

Violence against transgender (trans) women is widespread and systemic in the US. Trans people are not a protected class under the Constitution and trans rights often hinge on case law interpretations or individual state laws [1]. Policies such as those barring trans people from the military [2], supporting workplace discrimination against lesbian, gay, bisexual, transgender, and queer (LGBTQ) people [3], and permitting shelter discrimination against trans people, have made society more unsafe for trans people [4]. On the fourth anniversary of the Pulse nightclub massacre in Orlando, Florida—an anti-LGBTQ hate crime that took the lives of 49 people [5]— health nondiscrimination protections for transgender people were rolled back [6]. In the same week, two Black trans women were brutally murdered in the US [7]. Their names were Riah Milton and Dominique “Rem’Mie” Fells. There were among the 44 trans women, disproportionately Black and Latina, who were reportedly killed in 2020 [7]. This number is likely an underestimate due to police misgendering and underreporting of transgender status [8].

Trans women experience high rates of violence [9, 10], which creates risk for poor quality of life and adverse health outcomes [11–13], including post-traumatic stress disorder, suicide ideation, substance use, and HIV [14–16]. Gender-based violence (GBV)—violence targeting a person due to their actual or perceived gender—is one form of violence that transgender people commonly experience [9].

A growing number of studies have examined forms of GBV experienced by trans people and their health impact [9]. One form of GBV affecting trans people is transphobic hate crimes [17, 18]. A hate crime, as defined by the Federal Bureau of Investigations (FBI), is a criminal offense against a person or property, motivated in whole or in part by an offender’s bias against a race, religion, disability, sexual orientation, ethnicity, gender, or gender identity [19]. According to FBI’s data, approximately 16.8% of reported hate crimes in 2019 were based on sexual orientation bias and 2.1% on anti-transgender bias [20]. While, few legal protections exist for trans people, crimes committed on the basis of gender identity are covered by federal hate crime laws [21] and those of 18 states [22].

A systematic review article on violence motivated by sexual orientation and gender identity found that, among trans people, the prevalence of physical assault ranged from 11.8 to 68.2% and sexual assault from 7.0 to 49.1% [18]. Trans individuals reported experiencing sexual assault at twice the rate of cisgender LGBQ people in a study on lifetime experiences of sexual victimization [23]. Structural factors such as homelessness, poverty, unemployment, sex work, incarceration, and housing discrimination and instability have been associated with trans women’s risk for violence [9, 24–27]. Additionally, research suggests that people who are genderqueer or perceived as gender nonconforming are more likely to experience harassment and traumatic events than binary and cisgender people [15, 28, 29]. Thus, trans women’s risk of GBV may differ based on whether they are seen by others as the gender they identify with or what some term as “passing” [30].

Trans people of color, especially Black and Latina trans women, are at higher risk for hate crimes compared to their White counterparts due to the intersectional effects of racism and transphobia [10, 31]. For example, between 2012 to 2015, trans women made up 47 of the 88 anti-LGBTQ homicides recorded in 12 states; of these, 39 (44%) were Black trans women, underscoring a significant racial disparity in homicide rates [10]. Another study found that, from 2010 to 2014, young Black and Latina trans women had higher homicide rates than their cisgender woman counterparts [32]. These disparities suggest that racism and its sociocultural effects may place trans women of color at higher risk of violence.

Although hate crimes are common, they are generally underreported to the police [33]. Crime report rates are particularly low among trans people due to victimization by and discomfort with police, as well as a lack of faith in the police’s ability to effectively respond to hate crimes [17, 34]. Police report rates may be even lower for trans people of color due to racist policing policies and historic and ongoing distrust of the police in communities of color [26, 35, 36].

Despite the alarming rates of violence targeting trans women, a systematic review found that, globally, few studies (*n* = 15) have focused on violence perpetrated against trans people motivated by perception of their gender identity [18]. Most of the violence research that includes trans women has focused on intimate partner violence and sexual violence [9, 11] or reports older data on hate crime experiences [27]. There is a gap in the literature for population-based studies on hate crimes against trans women and their intersection with other factors affecting trans women (e.g., homelessness, immigration, racism, and incarceration). Additionally, little is understood about racial/ethnic variation in transphobic hate crime experiences among trans women and reporting them to the police. Such data are critically needed to inform interventions and to address inequities in the epidemic of violence towards trans women, especially trans women of color.

Our study aimed to contextualize risk of transphobic hate crimes by examining experiences, types, and correlates of transphobic hate crimes experienced by trans women in the San Francisco Bay Area (SFBA) site of a longitudinal cohort study called Trans*National. We also examined whether these hate crimes were reported to police and factors associated with reporting. We stratified our analyses by race/ethnicity to examine racial/ethnic differences in trans women’s experiences of hate crimes.

## Methods

### Study design and setting

Our study examined data collected from a cohort of trans women (*N* = 631) in the SFBA as part of the Trans*National study. Trans*National is a population-based, social epidemiological study that aimed to measure HIV incidence and risk factors for HIV acquisition among trans women in four cities worldwide: the San Francisco Bay Area (US), Sao Paulo (Brazil), Nanjing (China), and Windhoek (Namibia). Data collection occurred from 2016 to 2018. We analyzed baseline survey data on hate crime victimization (*N* = 629) from the SFBA site.

### Study sample and recruitment process

Study eligibility criteria were: 1) 18 years of age or older, 2) resident of the SFBA, 3) assigned male sex at birth and identify as a gender other than male, and 4) spoke English or Spanish. Long-chain peer-referral was used for recruitment. Study procedures are discussed in detail elsewhere [37]. The initial participants, or “seeds”, came from diverse backgrounds by race/ethnicity, education, and HIV status. Seeds were asked to recruit up to three self-identified trans women from their social network. For every successful recruit enrolled into the study, the participant recruiter received $20 USD. All participants provided informed consent. After consent was provided, interviewers administered surveys with a handheld computer. Participants received $50 USD for completing the survey.

### Measurements

Interviews were administered face-to-face by trained study staff at the study site. Measures were developed in conjunction with community advisors and while conducting formative research with trans women to identify constructs they find most relevant to their lives. Study measures assessed for this study were as follow:

#### Outcome variables

For the present analysis, there were two outcomes of interest: 1) self-reported experience of transphobic hate crimes (Have you ever been a victim of a transphobic hate crime?), which was reported as “yes/no”, and 2) report of transphobic hate crime incident to the police (Did you file a police report?), which was recorded as “none”, “some”, or “all”. For this analysis, report of transphobic hate crime incident to the police was dichotomized as ever/never. If answering yes to experiences of transphobic hate crimes, a further question was asked about what type, including robbery, physical assault, sexual assault, or battery with weapon specifically motivated by prejudice against trans people. Participants were able to select more than one type of hate crime.

#### Sociodemographic and other independent variables

Demographic variables were age, gender identity, sexual orientation (gay, bisexual, or straight), race/ethnicity, marital status, educational level, income, immigration, incarceration history, housing status, and sex work. Gender was categorized as female, transgender female/transwoman, genderqueer, and androgynous. Race/ethnicity was assessed using the US Census definitions of race/ethnicity and was categorized in our analysis as African American, Latina, White, or Multiracial/Other (which included Asian/Pacific Islander and Native American due to small sample sizes in these groups).

Ever undocumented as an immigrant, childhood and adult homelessness, and history of sex work were dichotomized (yes/no). Participants’ current housing was assessed as own house, renting a house/apartment/room, single room occupancy, homeless/temporary/unstable housing, or other. Incarceration history (number of times incarcerated) was dichotomized as yes if incarcerated one or more times and no if never. For our analysis, we used the following question from the *Experiences of Discrimination (EOD) Scale* [38, 39]*,* “Have you ever experienced discrimination, been prevented from doing something, or been hassled or made to feel inferior because of your gender identity or presentation, or race ethnicity, or color from the police or in the courts?” Participants who indicated “yes” were asked if this experience was related to gender identity or presentation, race/ethnicity, or both.

#### Gender identity passing

Gender identity passing (“How well do you feel you pass in society as cis-gender female?”) and gender identity questioning (“How often do you feel you are being clocked (or have your gender identity questioned)?”) were categorized based on a 4-point Likert scale (not at all, a little bit, somewhat, and a lot).

### Analysis

Analyses were conducted using Stata 16 (StatCorp. 2017, College Station: TX). Demographic characteristics were summarized using means for continuous variables, and proportions for categorical variables. Chi-squared analyses tested racial differences with experiences of transphobic hate crimes and police report of the crimes, hate crime type, experiences of discrimination by police/courts, incarceration, gender identity passing, and other independent variables. Bivariate logistic regression models assessed associations between independent variables (i.e., demographics, gender identity passing, immigration status) and each study outcome (‘ever experienced hate crimes’ and ‘reporting hate crime experiences to police’). Statistical significance was set at *P*-value < 0.05.

### Ethical approval

The Internal Review Board of the University of California San Francisco reviewed and approved the protocol for the study. Study methods were carried out in accordance with relevant ethical guidelines and regulations.

## Results

### Socioeconomic characteristics

There was a total of 629 trans women in the present study. The mean age of participants was 40.5 years (SD = 13) (Table 1). Most identified as trans women/trans female (48.3%) or women/female (47.3%) and most identified as straight/heterosexual (59.2%). Latina trans women were the largest racial/ethnic group (32.5%), followed by White (29.0%), Black/African American (17.0%), and other (Asian/Pacific Islander/Native American/multiracial) (21.5%); 12.6% of participants had ever been undocumented. Nearly one-third (30.4%) reported experiencing homelessness as a child and 72.6% reported experiencing homelessness as an adult. A majority of participants (64.7%) reported that they had ever engaged in sex work.

**Table 1:** Demographic characteristics of trans women surveyed in the San Francisco Bay Area, 2016–2018 (*N* = 629)

Variable	n (%)
**Age**	Mean = 40.5, SD = 13.0
18–24	70 (11.1)
25–34	169 (26.8)
35–44	144 (22.8)
45–54	140 (22.2)
55+	108 (17.1)
**Gender**	
Female	284 (47.3)
Transgender female/transwoman	290 (48.3)
Genderqueer/Genderfluid	23 (3.8)
Androgynous/Ambigender	3 (0.5)
**Sexual identity**	
Gay/homosexual	89 (19.5)
Bisexual	97 (21.3)
Straight/heterosexual	270 (59.2)
**Race**	
Black/African American	112 (17.0)
Latina	205 (32.5)
White	183 (29.0)
Other (Asian/Pacific Islander/Native American/multiracial)	136 (21.5)
**Marital Status**	
Never married	437 (69.3)
Married/living together as married	81 (12.8)
Divorced/separated	90 (14.3)
Widowed	23 (3.7)
**Educational level**	
Middle school or less	33 (5.2)
Some or completed secondary/GED	269 (42.8)
Some or completed college/Technical	292 (46.4)
Postgraduate	35 (5.6)
**Income per month**	
< $20,000	623 (99.8)
$20,000- $34,999	1 (0.2)
**Ever been undocumented**	
Yes	79 (12.6)
No	549 (87.4)
**Experienced homelessness as a child**	
Yes	192 (30.4)
No	439 (69.6)
**Experienced homelessness as an adult**	
Yes	458 (72.6)
No	173 (27.4)
**Current housing**	
Own home	17 (2.7)
Renting a house/apartment/room	273 (43.3)
Single room occupancy	120 (19.0)
Homeless/temporary/unstable housing	162 (25.7)
Other	59 (9.3)
**Ever did sex work**	
Yes	407 (64.7)
No	222 (35.3)

### Transphobic hate crimes and gender- and race/ethnicity-based abuse by race

A total of 45.8% of participants reported ever experiencing a transphobic hate crime (Table 2). Reporting transphobic hate crimes was highest among Latina (47.3%) and other race/ethnicity (47.8%) trans women, followed by Black (45.8%) and White (42.6%) trans women. Over half of participants (51.1%) who experienced a transphobic hate crime did not report the crime incident to the police.

**Table 2:** Experiences of transphobic hate crimes and criminal justice involvement, stratified by race/ethnicity, trans women surveyed in the San Francisco Bay Area, 2016–2018 (*N* = 629)

Variable	Total	Racial Group n (%)					
		White (***n*** = 183)	Black/AA (***n*** = 107)	Latina (***n*** = 205)	Other (***n*** = 136)	***χ***^**2**^	*p*-value
**Ever experienced transphobic hate crime**	288 (45.8)	78 (42.6)	49 (45.8)	97 (47.3)	64 (47.8)	1.1418	0.767
**Hate crimes prevalence overall**							
Robbery	10 (1.6)	0 (0.0)	4 (3.8)	5 (2.5)	1 (0.8)	29.7768	0.003
Physical Assault	111 (17.9)	34 (18.9)	15 (14.2)	31 (15.2)	31 (23.9)		
Sexual Assault	54 (8.7)	20 (11.1)	6 (5.7)	13 (6.4)	15 (11.5)		
Battery with weapon	104 (16.8)	21 (11.7)	23 (21.7)	47 (23.0)	13 (10.0)		
Did not experience hate crime	341 (55.0)	105 (58.3)	58 (54.7)	108 (52.9)	70 (53.9)		
**Type of hate crime among those who experienced a hate crime (*n*** **= 279)**						28.5007	0.001
Robbery	10 (3.6)	0 (0.0)	4 (8.3)	5 (5.2)	1 (1.7)		
Physical Assault	111 (39.8)	34 (45.3)	15 (31.3)	31 (32.3)	31 (51.6)		
Sexual Assault	54 (19.3)	20 (26.7)	6 (12.5)	13 (13.5)	15 (25.0)		
Battery with weapon	104 (37.3)	21 (28.0)	23 (47.9)	47 (49.0)	13 (21.7)		
**Mean number of hate crime types experienced (Std. Dev)**	1.9 (1.1)	1.8 (1.0)	2.0 (1.1)	2.1 (1.1)	1.5 (0.9)	NA	NA
**Reported hate crime to police (*n*** **= 286)**						7.3416	0.290
No, for none	146 (51.1)	42 (55.3)	24 (49.0)	43 (44.3)	37 (57.8)		
Yes, for some	46 (16.1)	15 (19.7)	5 (10.2)	17 (17.5)	9 (14.1)		
Yes, for all	94 (32.8)	19 (25.0)	20 (40.8)	37 (38.2)	18 (28.1)		
**Ever been incarcerated**						62.2038	< 0.001
No	230 (36.5)	102 (55.7)	12 (11.2)	77 (37.6)	39 (28.9)		
Yes	400 (63.5)	81 (44.3)	95 (88.8)	128 (62.4)	96 (71.1)		
**Ever experienced discrimination by police/in court (*n*** **= 324)**						59.2099	< 0.001
Based on gender identity/presentation	188 (58.0)	75 (91.5)	21 (34.4)	54 (50.0)	38 (52.1)		
Based on race, ethnicity, or color	10 (3.1)	0 (0.0)	1 (1.6)	6 (5.6)	3 (4.1)		
Based on both gender and race/ethnicity	126 (38.9)	7 (8.5)	39 (64.0)	48 (44.4)	32 (43.8)		

The mean number of hate crime types experienced was 1.9 (SD = 1.1). Physical assault was the most often reported transphobic hate crime type (39.8%), followed by battery with a weapon (37.3%), sexual assault (19.3%), and robbery (3.6%). The type of hate crime experienced differed significantly by race/ethnicity. Among those who experienced a transphobic hate crime, Latina (49.0%) and Black (47.9%) trans women had the highest prevalence of experiencing transphobic battery with a weapon, while White (26.7%) and other race/ethnicity (25.0%) trans women had a higher prevalence of transphobic sexual assaults (χ^2^ = 28.5, *p* = 0.001). Similarly, out of the overall sample, Latina (23.0%) and Black (21.7%) trans women had the highest prevalence of transphobic battery with a weapon, while White (11.1%) and other race/ethnicity (11.5%) trans women had the highest prevalence of transphobic sexual assaults.

There were significant differences between racial/ethnic groups regarding incarceration (Table 2). Incarceration rates were high among participants, with Black (88.8%), those categorized as other (71.1%), and Latina (62.4%) trans women reporting significantly higher incarceration rates than White (44.3%) trans women (χ^2^ = 62.2, *p* < 0.001). Among those who reported experiencing police/court discrimination, Black (64.0%) trans women were more likely to report experiencing discrimination based on both their gender and racial/ethnic identities, while White trans women (91.5%) were more likely to report experiencing discrimination based on gender identity/presentation alone compared to other racial/ethnic groups (χ^2^ = 59.2, *p* < 0.001).

Racial differences were also observed in participants’ perceptions of their gender identity and presentation (Table 3). A higher proportion of Black (51.4%) and Latina (49.3%) trans women reported that they felt their gender identity passed a lot compared to White (24.9%) and other (31.4%) trans women (χ^2^ = 41.2, *p* < 0.001). Conversely, a higher proportion of White (43.0%) and other (30.6%) trans women reported that they felt their gender identity was questioned a lot compared to Black (19.8%) and Latina (17.3%) trans women (χ^2^ = 43.5, *p* < 0.001).

**Table 3:** Experiences of gender identity presentation, stratified by race/ethnicity, trans women surveyed in the San Francisco Bay Area, 2016–2018 (*N* = 629)

Variable	Total	Racial Group n (%)					
		White (***n*** = 183)	Black/AA (***n*** = 107)	Latina (***n*** = 205)	Other (***n*** = 136)	***χ***^**2**^	***p***-value
**Feel gender identity passes**						41.1525	< 0.001
Not at all	59 (9.4)	26 (14.4)	7 (6.5)	10 (4.9)	16 (11.9)		
A little bit	97 (15.5)	38 (20.9)	11 (10.3)	28 (13.8)	20 (14.9)		
Somewhat	227 (38.7)	72 (39.8)	34 (31.8)	65 (32.0)	56 (41.8)		
A lot	242 (38.7)	45 (24.9)	55 (51.4)	100 (49.3)	42 (31.4)		
**Feel gender identity questioned**						43.4658	< 0.001
Not at all	70 (11.3)	7 (3.9)	18 (17.0)	29 (14.4)	16 (11.9)		
A little bit	210 (33.8)	54 (30.2)	35 (33.0)	79 (39.1)	42 (31.4)		
Somewhat	167 (26.9)	41 (22.9)	32 (30.2)	59 (29.2)	35 (26.1)		
A lot	174 (28.0)	77 (43.0)	21 (19.8)	35 (17.3)	41 (30.6)		

### Bivariate associations of transphobic hate crimes

Table 4 presents data on bivariate associations between transphobic hate crime experiences and socioeconomic indicators and experiences of gender and race/ethnicity-based abuse/harassment. There was an inverse dose-response relationship between educational attainment and experiencing a transphobic hate crime (*p* < 0.05). Trans women who experienced homelessness as a child (OR = 2.18; 95%CI: 1.54–3.08; *p* < 0.001) and an adult (OR = 3.01; 95%CI: 2.05–4.40; *p* < 0.001) had significantly higher odds of experiencing a transphobic hate crime compared to those who did not experience homelessness. Compared to trans women who owned their home, those who were living in single room occupancies (OR = 4.74; 95%CI: 1.29–17.37; *p* = 0.019), homeless or housing unstable (OR = 4.67; 95%CI: 1.29–16.86; *p* = 0.019), or in other housing situations (OR = 4.83; 95% CI: 1.25–18.57; *p* = 0.022) had significantly higher odds of experiencing a hate crime.

**Table 4:** Bivariate associations between experiencing a hate crime and demographic characteristics, trans women surveyed in the San Francisco Bay Area, 2016–2018 (*N* = 629)

Variable	Experienced transphobic hate crime n (%)		OR (95% CI)	***p***-value
	No (***n*** = 341)	Yes (***n*** = 288)		
**Age**				
18–24	42 (12.3)	28 (9.7)	REF	
25–34	97 (28.5)	71 (24.7)	1.10 (0.62, 1.94)	0.747
35–44	73 (21.4)	71 (24.7)	1.46 (0.82, 2.60)	0.201
45–54	67 (19.7)	72 (25.0)	1.61 (0.90, 2.89)	0.108
55+	62 (18.2)	46 (16.0)	1.11 (0.60, 2.05)	0.732
**Gender**				
Female	161 (50.0)	121 (43.8)	REF	
Transgender female/Trans woman	145 (45.0)	145 (52.6)	1.33 (0.96, 1.85)	0.089
Genderqueer/Genderfluid	15 (4.7)	8 (2.9)	0.71 (0.29, 1.73)	0.450
Androgynous/Ambigender	1 (0.3)	2 (0.7)	2.66 (0.24, 29.69)	0.426
**Sexual identity**				
Gay/Homosexual	55 (22.6)	33 (15.6)	REF	
Bisexual	50 (20.6)	46 (21.8)	1.53 (0.85, 2.76)	0.155
Heterosexual	138 (56.8)	132 (62.6)	1.59 (0.97, 2.61)	0.064
**Education**				
Middle school or less	11 (3.2)	22 (7.6)	REF	
Some or completed secondary	142 (41.6)	127 (44.1)	0.45 (0.21, 0.95)	0.039
Some or completed college/Technical	166 (48.7)	126 (43.8)	0.38 (0.17, 0.81)	0.012
Postgraduate	22 (6.5)	13 (4.5)	0.30 (0.11, 0.80)	0.017
**Ever been undocumented**				
No	305 (89.7)	244 (84.7)	REF	
Yes	35 (10.3)	44 (15.3)	1.57 (0.97, 2.53)	0.06
**Experienced homelessness as a child**				
No	263 (77.1)	175 (60.8)	REF	
Yes	78 (22.9)	113 (39.2)	2.18 (1.54, 3.08)	< 0.001
**Experienced homelessness as an adult**				
No	126 (36.9)	47 (16.3)	REF	
Yes	215 (63.1)	241 (83.7)	3.01 (2.05, 4.40)	< 0.001
**Current Housing**				
Own	14 (4.1)	3 (1.1)	REF	
Renting	158 (46.3)	114 (39.6)	3.37 (0.95, 11.99)	0.061
Single room occupancy (SRO)	59 (17.3)	60 (20.8)	4.74 (1.29, 17.37)	0.019
Homeless/temporary/unstable housing	81 (23.8)	81 (28.1)	4.67 (1.29, 16.86)	0.019
Other	29 (8.5)	30 (10.4)	4.83 (1.25, 18.57)	0.022
**Ever did sex work**				
No	163 (47.9)	59 (20.6)	REF	
Yes	177 (52.1)	228 (79.4)	3.56 (2.49, 5.08)	< 0.001
**Ever experienced discrimination by police/in court**				
Based on gender identity/presentation	66 (59.5)	121 (57.4)	REF	
Based on race, ethnicity, or color	5 (4.5)	5 (2.4)	0.55 (0.15, 0.35)	0.352
Based on both gender and race/ethnicity	40 (36.0)	85 (40.3)	1.16 (0.72, 1.87)	0.547
**Feel gender identity passes**				
Not at all	36 (10.6)	23 (8.1)	REF	
A little bit	48 (14.2)	49 (17.3)	1.60 (0.83, 3.08)	0.162
Somewhat	114 (33.6)	111 (39.1)	1.52 (0.85, 2.74)	0.158
A lot	141 (41.6)	101 (35.6)	1.12 (0.63, 2.01)	0.700
**Feel gender identity questioned**				
Not at all	50 (14.9)	20 (7.1)	REF	
A little bit	126 (37.5)	84 (29.7)	1.67 (0.93, 3.00)	0.088
Somewhat	81 (24.1)	85 (30.0)	2.62 (1.44, 4.79)	0.002
A lot	79 (23.5)	94 (33.2)	2.97 (1.63, 5.41)	< 0.001
**Ever been incarcerated**				
No	150 (44.1)	80 (27.8)	REF	
Yes	190 (55.9)	208 (72.2)	2.05 (1.47, 2.87)	< 0.001

Compared to those who did not have a history of sex work, trans women who had ever engaged in sex work (OR = 3.56; 95%CI: 2.49–5.08; *p* < 0.001) had greater odds of having experienced a hate crime. Similarly, those with a history of being undocumented (OR = 1.57; 95%CI: 0.97–2.53; *p* = 0.06) had significantly greater odds of experiencing a hate crime compared to those without a history of being undocumented. Additionally, those who felt their gender identity had been questioned somewhat (OR = 2.62; 95%CI: 1.44–4.79; *p* = 0.002) and a lot (OR = 2.97; 95%CI: 1.63–5.41; *p* < 0.001) had significantly greater odds of having experienced a hate crime compared to those who did not feel their gender identity was questioned. Finally, participants who reported a history of incarceration were significantly more likely to report experiencing a transphobic hate crime compared to those who did not report a history of incarceration (OR = 2.05; 95% CI: 1.47–2.87; *p* < 0.001).

### Bivariate associations for reporting transphobic hate crimes to police

Table 5 presents data on bivariate associations between reports of transphobic hate crimes to police and socioeconomic indicators, experiences of gender and race-based discrimination, and perception of gender identity and presentation. Those who were ever undocumented as an immigrant had greater odds of reporting a hate crime to police compared to those who were never undocumented (OR = 2.57; 95%CI: 1.29–5.09; *p* = 0.007). Trans women who reported experiencing discrimination by the police/in court based on both gender and race/ethnicity had greater odds of reporting a hate crime experience to police (OR = 2.45; 95%CI: 1.38–4.34; *p* = 0.002) compared to those who reported being discriminated against based solely on their gender identity/presentation.

**Table 5:** Bivariate associations between reporting hate crime to police and demographic characteristics, trans women surveyed in the San Francisco Bay Area, 2016–2018 (*N* = 286)

Variable	Reported transphobic hate crime to police n (%)		OR (95% CI)	***P-value***
	No (***n*** = 146)	Yes (140)		
**Age**				
18–24	14 (9.6)	13 (9.3)	REF	
25–24	43 (29.5)	28 (20.0)	0.70 (0.29, 1.71)	0.436
35–44	25 (17.1)	46 (32.9)	1.98 (0.81, 4.87)	0.136
45–54	40 (27.4)	32 (22.9)	0.86 (0.36, 2.09)	0.742
55+	24 (16.4)	21 (15.0)	0.94 (0.36, 2.45)	0.903
**Gender**				
Female	62 (45.3)	59 (43.1)	REF	
Transgender female/transwoman	67 (48.9)	76 (55.5)	1.19 (0.73, 1.94)	0.478
Genderqueer/Genderfluid	6 (4.4)	2 (1.4)	0.35 (0.07, 1.80)	0.210
Androgynous/Ambigender	2 (1.5)	0 (0.0)	–	
**Sexual identity**				
Gay/homosexual	14 (14.4)	19 (16.8)	REF	
Bisexual	29 (29.9)	16 (14.2)	0.41 (0.16, 1.02)	0.056
Heterosexual	54 (55.7)	78 (69.0)	1.06 (0.49, 2.30)	0.874
**Race**				
White	42 (28.8)	34 (24.3)	REF	
Black/African American	24 (16.4)	25 (17.8)	1.29 (0.63, 2.64)	0.492
Latina	43 (29.5)	54 (38.6)	1.55 (0.85, 2.84)	0.154
Other	37 (25.3)	27 (19.3)	0.90 (0.46, 1.76)	0.762
**Education**				
Middle school or less	7 (4.8)	15 (10.7)	REF	
Some or completed secondary	69 (47.3)	57 (40.7)	0.38 (0.15, 1.01)	0.052
Some or completed college/Technical	60 (41.1)	65 (46.4)	0.51 (0.19, 1.32)	0.165
Postgraduate	10 (6.8)	3 (2.2)	0.14 (0.03, 0.67)	0.014
**Ever been undocumented**				
No	132 (90.4)	110 (78.6)	REF	
Yes	14 (9.6)	30 (21.4)	2.57 (1.29, 5.09)	0.007
**Experienced homelessness as a child**				
No	95 (65.1)	79 (56.4)	REF	
Yes	51 (34.9)	61 (43.6)	1.44 (0.89, 2.32)	0.135
**Experienced homelessness as an adult**				
No	23 (15.8)	23 (16.4)	REF	
Yes	123 (84.2)	117 (83.6)	0.95 (0.51, 1.79)	0.877
**Current Housing**				
Own	1 (0.7)	2 (1.4)	REF	
Renting	59 (40.4)	54 (38.6)	0.46 (0.04, 5.19)	0.528
Single room occupancy	28 (19.2)	32 (22.9)	0.57 (0.05, 6.65)	0.655
Homeless/temporary/unstable housing	40 (27.4)	41 (29.3)	0.51 (0.04, 5.88)	0.591
Other	18 (12.3)	11 (7.8)	0.31 (0.02, 3.78)	0.355
**Ever did sex work**				
No	29 (20.0)	29 (20.7)	REF	
Yes	116 (80.0)	111 (79.3)	0.96 (0.54, 1.70)	0.881
**Ever experienced discrimination by police/in court**				
Based on gender identity/presentation	71 (68.3)	48 (45.7)	REF	
Based on race, ethnicity, or color	1 (0.9)	4 (3.8)	5.92 (0.64, 54.57)	0.117
Based on both gender and race/ethnicity	32 (30.8)	53 (50.5)	2.45 (1.38, 4.34)	0.002
**Feel gender identity passes**				
Not at all	17 (11.8)	6 (4.4)	REF	
A little bit	25 (17.4)	24 (17.4)	2.72 (0.92, 8.06)	0.071
Somewhat	58 (40.3)	51 (36.9)	2.49 (0.91, 6.80)	0.075
A lot	44 (30.5)	57 (41.3)	3.67 (1.34, 10.08)	0.012
**Feel gender identity questioned**				
Not at all	6 (4.2)	14 (10.2)	REF	
A little bit	38 (26.6)	46 (33.3)	0.52 (0.18, 1.48)	0.220
Somewhat	47 (32.9)	37 (26.8)	0.34 (0.12, 0.96)	0.042
A lot	52 (36.3)	41 (29.7)	0.34 (0.12, 0.96)	0.041
**Ever been incarcerated**				
No	45 (30.8)	35 (25.0)	REF	
Yes	101 (69.2)	105 (75.0)	1.34 (0.80, 2.25)	0.274

Participants’ perception and experiences of their gender identity and presentation were significant factors in whether they reported transphobic hate crime experiences to police. Trans women who felt their gender identity passes a lot (OR = 3.67; 95%CI: 1.34–10.08; *p* = 0.012) had greater odds of reporting a hate crime experience to the police compared to those who did not feel their gender passed at all. Conversely, trans women who felt their gender identity was questioned somewhat (OR = 0.34; 95%CI: 0.12–0.96; *p* = 0.042) and a lot (OR = 0.34; 95%CI: 0.12–0.96; *p* = 0.041) had lower odds of reporting a hate crime experience to the police compared to those who did not feel their gender identity was questioned at all.

## Discussion

Our study offers important data on a significant social crisis facing trans people in the U.S. and globally—that of pervasive violence rooted in the social marginalization and policing of trans people’s bodies and lives. We found that a high proportion of trans women in our study had experienced transphobic hate crimes, with significant socio-structural risk factors and racial/ethnic differences by crime type, suggesting that hate crime experiences may vary by racial group and other social categories. Transphobic hate crime estimates from our study contribute data that can help researchers and advocates address factors that shape hate crime victimization among trans people.

Nearly half of participants reported experiencing a transphobic hate crime in their lifetime, with Latina and other (Asian/Pacific Islander, Native American, multiracial) trans women reporting the highest proportions of hate crimes. Our estimate falls near the midpoint of the wide range reported by a review article on lifetime experiences of GBV (7–86%) among trans people [9] and is higher than the estimate of lifetime experiences of hate crime victimization (33.6%) among an urban LGBT sample [40]. Although not statistically significant, the higher prevalence of hate crime experiences among Latina and other trans women suggests that there might be social and environmental factors that place these women at higher risk of hate crimes and should be further explored in future research.

A key study finding is that transphobic hate crime incidents are underreported to the police across all racial groups, with more than half of participants not reporting hate crime experiences to the police, consistent with other studies [26, 40, 41]. The low rate of police reporting may be due to fear of further victimization through discrimination by police as other studies have found [26, 34]. Although our data reflect lifetime experiences of transphobic hate crimes, the low number of anti-trans incidents (*n* = 151) relative to all single-bias incidents (*n* = 7103) reported in 2019 [42] suggests that, nationally, these crimes are underreported to the police, as FBI hate crime reports suggest [43].

Although there were no significant racial differences in the overall hate crime rate, an important finding is that there were significant racial/ethnic differences in the types of hate crimes trans women experienced. Participants reported experiencing an average of two types of hate crimes. Among those who experienced transphobic hate crimes, and in the overall sample, Black and Latina trans women had the highest burden of battery with a weapon while White and other trans women had the highest burden of sexual assault. The higher prevalence of battery with a weapon—a form of violence that could be more lethal—experienced by Black and Latina trans women might partly explain the higher rate of fatal violence experienced by these two groups [7, 32]. These differences in hate crime type suggest that there may be racially distinct social conditions disproportionately predisposing some trans women to certain types of transphobic violence. For example, research examining state-level firearm data in the U.S. over a 25-year span found that racial residential segregation was positively associated with Black firearm homicides [44]. Future trans-led ethnographic and mixed-methods studies are needed to better understand the contexts in which transphobic hate crimes are experienced and the diverse factors that shape risk for different types of hate crimes.

Our study also found that various structural factors were significantly associated with greater odds of experiencing a transphobic hate crime: lower educational attainment, housing instability, homelessness as a child and an adult, a history of sex work, incarceration, and being undocumented. Housing instability is particularly important to highlight among trans women in the SFBA. Due to increased housing costs and gentrification, homelessness and housing instability have soared in the region, with trans women of color disproportionately impacted [45]. The association of housing with experiences of transphobic violence contributes to a growing body of literature linking homelessness and housing instability to various risks and outcomes such as HIV and drug use [46–48]. Moreover, the link between homelessness as a child and transphobic violence suggests that homelessness in childhood may have a persistent effect of placing trans women at higher risk of violence over their life course. Further studies should examine the relationship between housing and violence among trans women over the life course.

Similarly, the association between a history of undocumented immigration status and experiencing a transphobic hate crime is an important one that requires further examination. This finding is consistent with other studies showing a link between immigration status and experiences of violence among LGBT populations [49, 50]. Additionally, a qualitative study found that a lack of immigration documentation prohibited legal gender marker change [51], which has been associated with increased risk of GBV [9]. Other research has found a link between precarious documentation status and GBV experiences among immigrant women [52], suggesting there might be structural barriers associated with being undocumented that may predict GBV among trans women. Given the devastating impact of immigration policies on immigrant communities in the US over the past decade [53], immigration as a social determinant of violence among trans immigrants is a topic of research that may provide further insights into the health risks of lacking legal documentation.

Another notable finding is that trans women who reported both gender- and race-based discrimination by the police or court were more likely to report a hate crime experience to the police. This seems counterintuitive and without establishing temporality, we are unable to determine whether the experiences of discrimination were an outcome of reporting a transphobic hate crime or preceded the incident. Future longitudinal studies could help answer the directionality of the association and how the intersection of racial and gender identities may shape trans women’s experiences of the legal system. Another surprising finding is that trans women who were ever undocumented were significantly more likely to report a hate crime compared to those who were documented. One study found that Latina trans women reported that they avoided encounters with the police while undocumented, however, after receiving documentation, services and legal gender marker change were accessible to them and fear of deportation was reduced [51]. Our measure was only a history of being undocumented; thus, we cannot assess whether a change in our participants’ documentation status made participants more comfortable to report to the police compared to those who were never undocumented. Additionally, San Francisco has been a long time sanctuary city [54]—a municipal area that limits its cooperation with federal enforcement of immigration laws. Thus, undocumented immigrants may feel more comfortable reporting hate crime experiences to the police.

Gender presentation and perceived gender also factored into whether trans women experienced a transphobic hate crime and whether they reported the crime incident to police. Trans women who felt that their gender identity had been questioned were significantly more likely to experience a hate crime but less likely to report the incident to police compared to those who did not feel their gender was questioned. Conversely, trans women who perceived that they “passed” as women were more likely to report hate crime incidents to the police, compared to those who did not think they passed. This finding suggests that hate crime experiences of trans women who feel they do not pass as their gender or who have had their gender questioned are likely underreported. Passing may be motivated by internal and external validation and fear of gender discrimination and further victimization by police [55], which may explain the low reporting rate. Other studies have found that gender nonconformity can lead to experiencing prejudice among lesbian, gay, and bisexual people [28], but none to our knowledge have shown an association between “passing” and experiencing a hate crime.

### Limitations

There are study limitations that require noting. First, we did not collect data on whether participants’ police reporting patterns differed by the type of hate crime they experienced. Disaggregated data on hate crime type and police reporting could offer important insights into how the type of violence trans women experience shape their willingness to report to the police. For example, prior research suggest that sexual assault is underreported compared to other crimes [56].

Another limitation is that we did not provide a definition of transphobic hate crimes to participants. Rather, participants were asked to indicate whether they have experienced such incidents based on their perception. This may lead to internal validity issues and bias towards over- or under-reporting of hate crimes. Third, our study was an exploratory analysis of an understudied issue, thus, we only conducted bivariate analysis of dependent and independent variables to identify factors associated with transphobic hate crime experiences and police reporting. Future studies should further explore significant factors to assess which are independently associated with the two study outcomes. Fourth, we did not establish temporality in our study since we analyzed cross-sectional baseline data. For example, as noted above, it is not clear whether trans women experienced race- and gender-based discrimination by police and the courts while reporting a hate crime experience or prior to reporting. Fifth, the collapsing of multiple racial categories (Asian/Pacific Islander, Native American, and multiracial/other) into one, “other”, due to small number of observations for each group limits our ability to determine differences in experiences and risk factors of individual racial groups within this category. Sixth, hate crime types were not mutually exclusive; thus, denominators for the bivariate analysis testing racial/ethnic differences do not perfectly sum to racial/ethnic category totals, however they are close. Finally, our data were collected in the SFBA only, therefore limiting generalizability. Nonetheless, the SFBA is home to trans people from other areas of the U.S. and, although not recorded in the present study, lifetime experiences may have occurred in other locations.

## Recommendations

Trans lives matter, yet high levels of violence targeting trans people like the women in our study indicate that this is not currently a reality in the US. Structural factors associated with social marginalization and HIV among trans women were among the main factors associated with experiencing transphobic hate crimes [12]. Our findings direct researchers, policymakers, and advocates to take measures to address the different ways trans women experience GBV and further research the contextual factors that may be shaping these different experiences.

Policy and program interventions should be developed to address structural conditions that increase trans women’s risk of experiencing GBV, with critical attention to racial differences and disparities. Given that factors such as immigration history, sex work, and housing are associated with experiencing a transphobic hate crime, and given that nearly all of the women in the study lived at or below the poverty line, reforms that provide trans women with immigration documentation, free or affordable housing, income support, and work opportunities are critical to helping improve the contextual factors that exacerbate their risk of violence. Additionally, due to inadequate historical and current support from local governments, trans communities in the SFBA have built advocacy organizations and networks to support themselves. However, more structural and financial support of these organizations is needed to address continued inequities facing trans community members. Also critically important, our finding that those whose gender was questioned were more likely to experience a hate crime but less likely to report it to police highlights the critical need for structural changes that ensure medical, social, and legal gender affirmation for trans people.

Additionally, the low level of police reporting of transphobic hate crimes suggests that trans women might be disillusioned with law enforcement’s ability to bring justice to trans survivors of violence due to experiences of police disbelief and prejudice [26]. Amidst the ongoing movement for Black lives’ push to radically rethink the notion of policing as an institution that brings safety or justice [57], alternatives to reporting hate crimes to the police are urgently needed. Alternative approaches that emphasize care and safety, such as community-based emergency first responder methods, are gaining more attention and support [58]. These include community-based organizations that offer crisis response and other social services, as well as restorative [59] and transformative justice approaches from an intersectional feminist framework [60]. Additionally, establishing a department in municipal governments where victims of violence can report their experiences as an alternative to the police may address the underreporting of violence against trans people and improve our understanding of the scale of this crisis.

Finally, anti-violence, trans advocacy, and social justice groups have put forward various demands and policy recommendations on how to address violence against trans people that should be engaged with and implemented, including the San Francisco LGBTQ violence prevention needs assessment recommendations and the Trans Agenda for Liberation [61–63]. Recommendations include decriminalization of trans people; an end to structural, symbolic, and physical forms of transphobic violence and the social conditions that perpetuate these; and inclusion of gender identity in the Civil Rights Act. The implementation of these demands are critical to enabling systemic and social transformation of gender hierarchies and norms that shape transphobic violence, as trans activist, Raquel Willis, has astutely argued for [64], and to address related socioeconomic inequities in order to create safer conditions for trans people.

## Data Availability

Data for this study can be requested from author, Erin C. Wilson.

## Abbreviations

GBV: Gender-based violence
SFBA: San Francisco Bay Area
